# Agriculture causes nitrate fertilization of remote alpine lakes

**DOI:** 10.1038/ncomms10571

**Published:** 2016-02-08

**Authors:** E. J. Hundey, S. D. Russell, F. J. Longstaffe, K. A. Moser

**Affiliations:** 1 Department of Geography, The University of Western Ontario, Social Science Centre Room 2322, 1151 Richmond Street, London, Ontario, Canada N6A 5C2; 2 Department of Earth Sciences, The University of Western Ontario, Biological and Geological Sciences Building, 1151 Richmond Street, London, Ontario, Canada N6A 5B7

## Abstract

Humans have altered Earth's nitrogen cycle so dramatically that reactive nitrogen (Nr) has doubled. This has increased Nr in aquatic ecosystems, which can lead to reduced water quality and ecosystem health. Apportioning sources of Nr to specific ecosystems, however, continues to be challenging, despite this knowledge being critical for mitigation and protection of water resources. Here we use *Δ*^17^O, *δ*^18^O and *δ*^15^N from Uinta Mountain (Utah, USA) snow, inflow and lake nitrate in combination with a Bayesian-based stable isotope mixing model, to show that at least 70% of nitrates in aquatic systems are anthropogenic and arrive via the atmosphere. Moreover, agricultural activities, specifically nitrate- and ammonium-based fertilizer use, are contributing most (∼60%) Nr, and data from other North American alpine lakes suggest this is a widespread phenomenon. Our findings offer a pathway towards more effective mitigation, but point to challenges in balancing food production with protection of important water resources.

## Abstract (web summary)

The long-range atmospheric transport and deposition of reactive nitrogen may be affected by human activities. Here, the authors use isotope data to constrain sources of reactive nitrogen to high elevation lakes in the Uinta Mountains, finding that the majority originates from distant agricultural activities.

## Graphical abstract

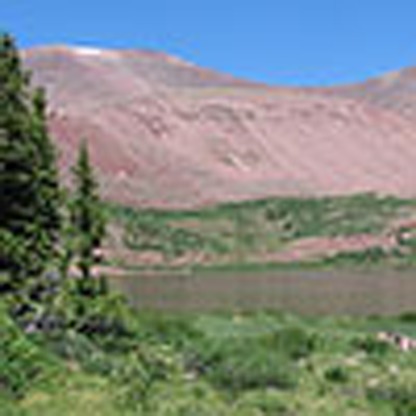

The global nitrogen cycle has been drastically altered by human activities^1^,^2^. Reactive nitrogen (Nr), or biologically available nitrogen, which includes the inorganic forms ammonia (NH_3_), ammonium (NH_4_^+^), nitrogen oxide (NO_*x*_), nitric acid (HNO_3_), nitrous oxide (N_2_O), nitrate (NO_3_^−^) and others (for example, peroxyacetyl nitrates, as well as organic compounds (for example, urea, amines, proteins and nucleic acids)), has been doubled by human activities^3^. Nr is added to the environment naturally by lightning (5 Tg N per year) and by terrestrial (58 Tg N per year) and marine (140 Tg N per year) biological nitrogen fixation for a total of 203 Tg N per year^4^. Anthropogenic activities, including production of synthetic fertilizers and byproducts of fossil fuel combustion (NO_*x*_), add 210 Tg N per year of Nr (ref. 4). Terrestrial Nr emissions are estimated to be 100 Tg N per year (75 Tg N per year are anthropogenic)^4^, which, despite relatively short residence times, can be transported and deposited hundreds to thousands of kilometres from their source^5^,^6^.

Owing to the transportable nature of Nr, even remote regions may receive increased inputs^7^. Because of a paucity of monitoring stations in isolated areas, the relative contribution of atmospheric Nr is unclear and its specific sources are uncertain. Alpine lakes can be limited by nitrogen^8^,^9^,^10^,^11^ and thus even modest increases in nitrogen deposition can have significant effects including eutrophication, acidification and reduction of biodiversity^12^. To protect alpine lake ecosystems, which are globally important water resources for adjacent lowlands^13^ and biodiversity hotspots^14^, identification of the specific contributions of different nitrogen sources to these remote regions is needed to implement successful mitigation policies.

Here we use stable isotope analysis of nitrates (*δ*^15^N, *δ*^18^O and *Δ*^17^O) in modern water (inflows and lakes) and snow samples in combination with a stable isotope mixing-model (SIAR, Stable Isotope Analysis in R)^15^, to determine the proportional contribution of various nitrate sources to the Uinta Mountains, Utah, USA. This mountain range, located in northeast Utah (40° to 41° N, 109° to 111° W), has experienced little direct human impact, making it ideal for investigating the influence of atmospheric nitrate^16^. The ‘triple oxygen isotope' approach for the analysis of nitrate allows us to take advantage of the anomalous enrichment of ^17^O–NO_3_^−^ that results from transfers of ^17^O from ozone during photochemical conversion of NO_*x*_ to NO_3_ in the atmosphere^17^. This signature of a mass-independent oxygen isotope fractionation, quantified by the term *Δ*^17^O (*Δ*^17^O=*δ*^17^O−0.52 × *δ*^18^O), differs from the mass-dependent relationship between *δ*^17^O and *δ*^18^O characteristic of terrestrial processes, which is described by the terrestrial fractionation line. Therefore, using *Δ*^17^O–NO_3_^−^ allows for clear differentiation of nitrate originating from fossil fuel burning, biomass burning and lightning that is oxidized in the atmosphere versus nitrate oxidized in freshwater or terrestrial systems. The ^17^O anomaly is measurable after wet or dry deposition of atmospherically oxidized (and ^17^O-enriched) NO_3_^−^ at the Earth's surface and through subsequent reactions affecting this nitrate (for example, assimilation and denitrification)^18^. Analysis of *δ*^15^N and *δ*^18^O provides further insight into the sources and/or processes that control nitrate isotopic composition^19^,^20^,^21^,^22^. The *δ*^15^N is helpful in distinguishing various sources of nitrogen and, together with *δ*^18^O, can also be used to help identify reactions such as denitrification^23^. Values of *δ*^15^N–NO_3_^−^ have been used similarly in previous research to classify sources^24^,^25^,^26^. We have quantified the contributions of particular nitrate sources to inflow and lakewater samples using a stable isotope mixing model contained in the software package SIAR^27^,^28^. SIAR is particularly useful for nitrate source allocation for three reasons. First, uncertainty in the system can be included by factoring in variability in source values. Second, solutions can be found for systems with more potential sources than previous approaches have allowed. Third, probability distributions are generated for each source^15^.

Our results show that atmospheric deposition of Nr from anthropogenic sources contributes at least 70% of the total nitrate to Uinta Mountain inflows and lakes; the majority of this is from atmospherically delivered fertilizers. Similarities in nitrate isotope compositions (*δ*^15^N and *δ*^18^O) between Uinta Mountain lakes and lakes throughout the US Rocky Mountains suggest that these findings apply to other mountain regions in western North America. Our research underlines the importance of careful management of fertilizer application in adjacent lowlands to protect alpine ecosystems and also demonstrates the power of applying a combined nitrogen- and triple oxygen-isotope approach to quantify human inputs to the nitrogen cycle.

## Results

### Water chemistry and stable isotope compositions

**Figure 1:**
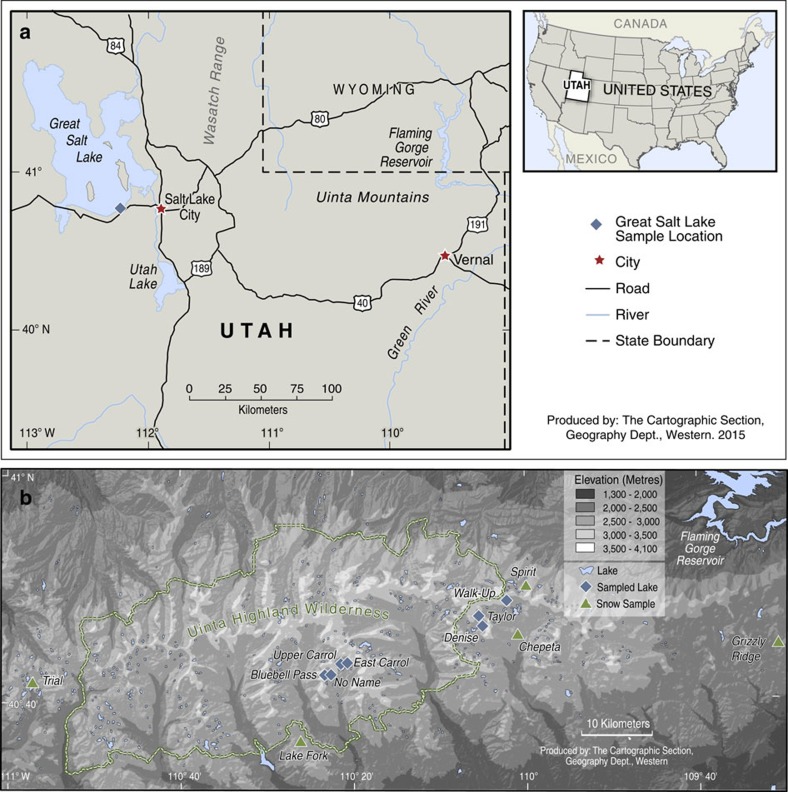
Map of Uinta Mountain study area. (**a**) Location of the Uinta Mountains in northeastern Utah and USA, including water sampling point in Great Salt Lake (see Supplementary Methods); (**b**) the locations of study lakes and snow sampling sites within the Uinta Mountains. Reproduced and modified from Hundey *et al*.^16^ with permission from John Wiley and Sons.

**Figure 2:**
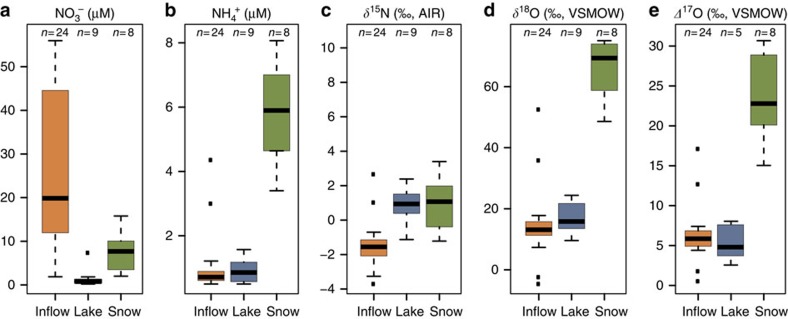
Summary boxplots of Uinta Mountain sample data. (**a**) NO_3_^−^ (μM), (**b**) NH_4_^+^ (μM), (**c**) *δ*^15^N-NO_3_^−^ (‰), (**d**) *δ*^18^O-NO_3_^−^ (‰) and (**e**) *Δ*^17^O-NO_3_^−^ (‰). The median is represented by the horizontal black bar, the lower and upper boundaries of the box represent the lower (25%) and upper (75%) quartiles of the data and the whiskers represent the minimum and maximum values, excluding outliers. Outliers are represented as points outside the whiskers. The NO_3_^−^ sample concentrations reported here are higher than the true average for these Uinta Mountain sites, because sample selection for isotopic analysis was limited by nitrate concentration.

**Figure 3:**
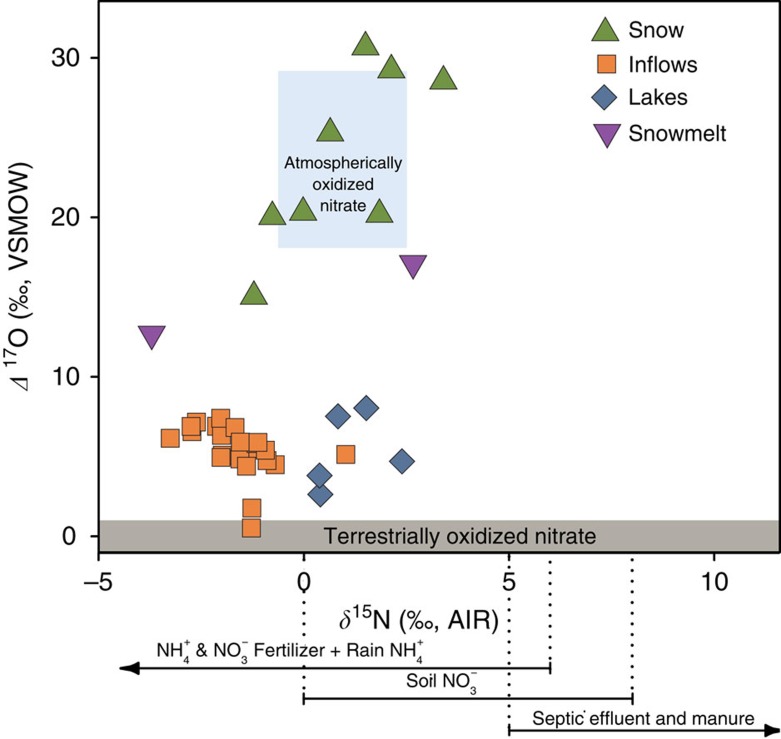
*Δ*^17^O-NO_3_^−^ versus *δ*^15^N-NO_3_^−^ of Uinta Mountain water samples relative to possible sources. The SIAR model inputs for nitrate sources are represented by the blue and grey boxes. The lines shown below the *x* axis (*δ*^15^N) indicate source N isotopic ranges (see also Supplementary Table 2). The overlap in source values for *δ*^15^N (NH_4_^+^ & NO_3_^−^ Fertilizer+Rain NH_4_^+^, Soil NO_3_^−^ and Septic Effluent and Manure) is shown by the overlap of lines below the *x* axis. Model inputs for the nitrate sources are drawn from the literature^21^ other than for AON, which was derived from the mean and s.d. of the Uinta Mountain snow samples. Samples plotting above terrestrial source *Δ*^17^O (0±1‰) contain a significant contribution from AON.

**Figure 4:**
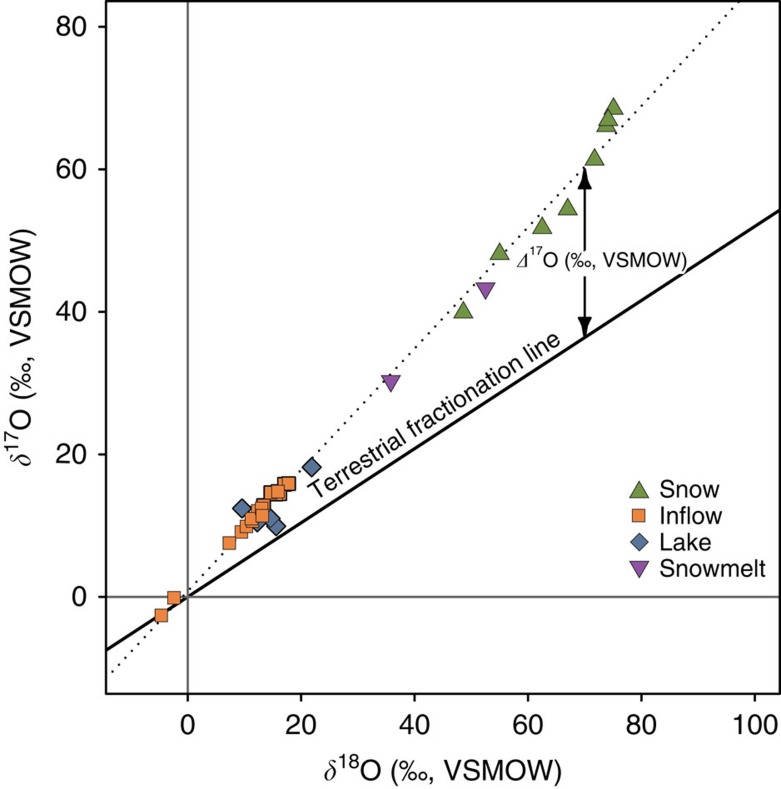
*δ*^17^O- versus *δ*^18^O-NO_3_^−^ of Uinta Mountain water samples compared with the terrestrial fractionation line. *Δ*^17^O is the difference between the measured *δ*^17^O and the *δ*^17^O expected based on the terrestrial fractionation line (*δ*^17^O=0.52 × *δ*^18^O)^56^.

Water chemistry and stable isotope compositions were measured in six Uinta Mountain lakes and their inflows and snow from nearby sites (Fig. 1). Mean values of *δ*^15^N–NO_3_^−^ for lakes (+0.8‰) and snow (+0.9‰) are higher than for inflows (−1.6‰) and *δ*^15^N–NO_3_^−^ of snow spans a slightly larger range than inflow and lake samples (range for snow is −1.2 to +3.4‰; for lakes is −1.1 to +2.4‰ and for inflows is −3.3 to +1.0‰) (Figs 2 and 3, and Supplementary Table 1). Inflows have higher and more variable nitrate concentrations (mean NO_3_^−^=28.7±16.78 μM) than do lakes (mean NO_3_^−^=1.6±2.2 μM), whereas mean NH_4_^+^ concentrations for inflows and lakes are identical (0.9 μM) (Fig. 2 and Supplementary Table 1). Mean values of *δ*^18^O–NO_3_^−^ for lakes (+16.7‰) are higher than for inflows (+11.7‰) (Fig. 2 and Supplementary Table 1). Of 41 samples analysed here, all but one have *Δ*^17^O–NO_3_^−^ that are elevated above the terrestrial fractional line (*Δ*^17^O=0±1‰) (Figs 3 and 4). Snow samples have particularly high *Δ*^17^O–NO_3_^−^ with a mean value of +23.7‰ (Figs 2 and 3, and Supplementary Table 1). Snow samples are also characterized by high mean NH_4_^+^ (5.8 μM) concentrations and *δ*^18^O–NO_3_^−^ (+66.0‰) (Fig. 2).

### Source contribution model

Potential sources of Nr to Uinta Mountain lakes and inflows are both distal and proximal (that is, within the catchment). Distal sources could include fossil fuel combustion from communities in the adjacent Uinta Basin and nearby (∼150 km) Wasatch Front and agriculture (including application of synthetic fertilizers, manure and urea) along the Wasatch Front or further upwind (for example, California) (Fig. 1). Catchment sources could include naturally biologically fixed nitrogen from soils or inflows and, although probably insignificant, manure from grazing sheep and cattle, which would have peaked in these catchments at the onset of the twentieth century^16^. Here we distinguish among four categories of sources for nitrates (Fig. 3 and Supplementary Table 2). The first is atmospherically oxidized nitrate (AON), which originates from natural (lightning, wildfire and soil emissions), anthropogenic (fossil fuel combustion and biomass burning) or mixed (for example, soil emissions from fertilized fields) sources that are oxidized atmospherically. The second is ‘NH_4_^+^ and NO_3_^−^ Fertilizer+Rain NH_4_^+^'. This category includes NO_3_^−^- and NH_4_^+^-based synthetic fertilizers and rain NH_4_^+^, which are combined because their sources are isotopically indistinguishable^21^. Rain NH_4_^+^ is largely derived from fertilizer, although natural soil emissions and manure can also contribute^21^,^29^. The third category is soil nitrate (Soil NO_3_^−^), which largely represents unamended soil from natural systems, for example, beneath forest vegetation^21^. The final is ‘Septic Effluent and Manure'.

**Figure 5:**
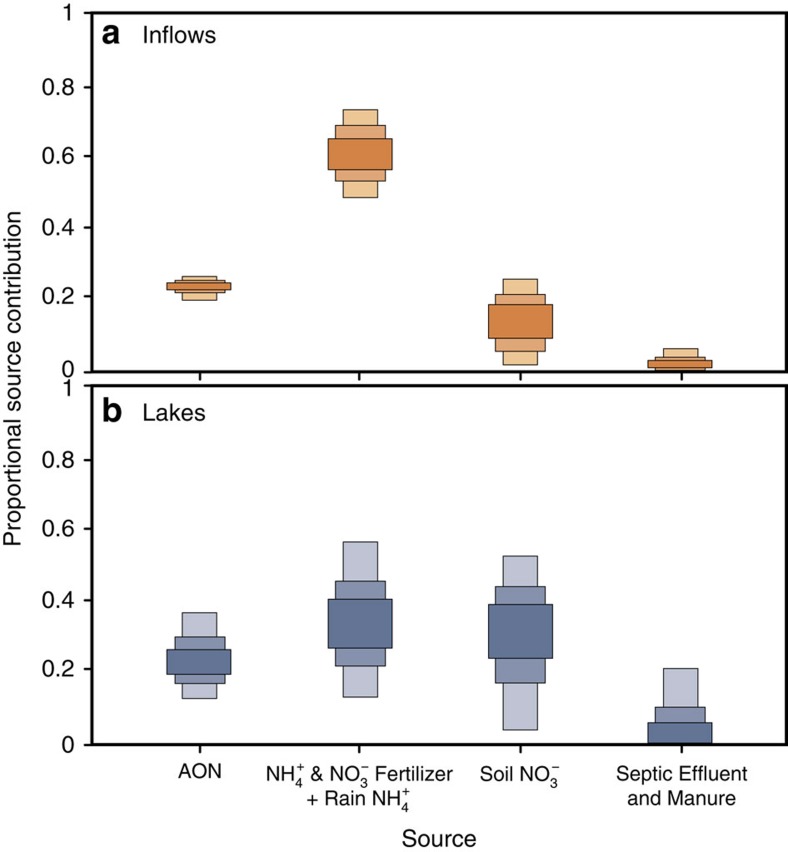
Mixing model estimates of nitrate source contributions. Mixing model estimates of contributions of nitrate sources based on *δ*^15^N-NO_3_^−^ and *Δ*^17^O-NO_3_^−^ of samples are displayed for (**a**) inflows and (**b**) lakes. Bayesian credible intervals show estimated contributions of each source to inflows and lakes, as determined using the stable isotope mixing model SIAR. The 50, 75 and 95% Bayesian credible intervals for each source are shown by the dark, medium and light boxes, respectively.

The proportional contribution of these sources to inflows and lakes are graphically summarized by SIAR using 95, 75 and 50% Bayesian credible intervals (Fig. 5). For inflows, NO_3_^−^ and NH_4_^+^ Fertilizer+Rain NH_4_^+^ is the largest contributor of nitrate, at 62% modal probability estimate (MPE, the solution with the highest probability) (Fig. 5 and error terms in Table 1). AON contributes 23% MPE to the total nitrate in inflows, Soil NO_3_^−^ contributes 14% MPE and Septic Effluent and Manure contributes 1% MPE (all *P*-values<0.01). NH_4_^+^- and NO_3_^−^-based fertilizers+rain NH_4_^+^ is also the main contributor to lake nitrate (35% MPE), although a much greater proportional contribution comes from Soil NO_3_^−^ (34% MPE) than for inflows. AON contributes 24% MPE to lakes, only slightly higher than inflows, and Septic Effluent and Manure contributes 2% MPE (Fig. 5). It is noteworthy that the Bayesian credible intervals for inflows are smaller compared with those for lakes, in particular for sources other than AON.

**Figure 6:**
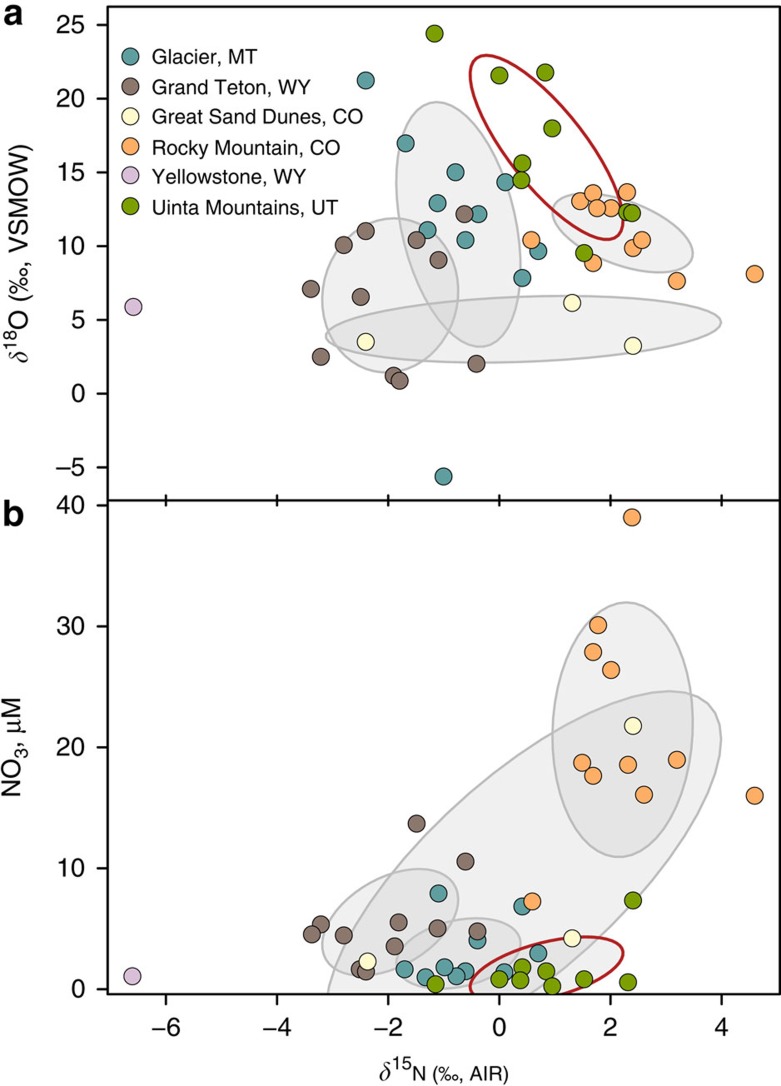
Comparison of Uinta Mountain lake nitrate sample isotopic compositions from five national parks in the US Cordillera. (**a**) *δ*^18^O- versus *δ*^15^N-NO_3_^−^ and (**b**) NO_3_^−^ concentration versus *δ*^15^N-NO_3_^−^. The ellipses, which outline the 50% confidence level of each location (grey outline for Rocky Mountain sites, red for Uinta Mountains), are used to visualize each group of lakes and the overlap among the sites. Yellowstone National Park is represented by a single measurement. Data for sites other than the Uinta Mountains are from Nanus *et al*^30^.

A comparison of the isotopic compositions of Uinta Mountain lake nitrates with those from national parks of the North American Cordillera^30^ shows similarity in lakewater NO_3_^−^ concentrations and *δ*^15^N- and *δ*^18^O–NO_3_^−^ isotopic compositions (Fig. 6). This suggests that our results may be applicable to other western US mountain ranges. If so, this would show that a large proportion of alpine Nr is arriving atmospherically from fertilizers.

## Discussion

Snow sample *δ*^15^N–NO_3_^−^ is higher and spans a greater range than inflow samples (Figs 2 and 3). To explain this feature, we hypothesize that some fertilizer-derived N is still present as NH_4_^+^ in the snowpack, where nitrification does not occur^31^. This is supported by the high concentrations of NH_4_^+^ in snow samples relative to the inflows and lakes (Fig. 2), and is further corroborated by a calculated snow *δ*^15^N–NH_4_^+^ of −4.5‰, which is within the known range for fertilizer ammonium (see Methods). On release from snow into soil, snowmelt or inflows, NH_4_^+^ can be nitrified to NO_3_^−^, resulting in the high NO_3_^−^ concentrations and lower *δ*^15^N–NO_3_^−^ that characterize inflows (Fig. 2).

There are several possible explanations for the higher *δ*^15^N–NO_3_^−^ in lakes relative to inflows, including atmospheric acidity, denitrification, differences in nitrate sources and/or nitrate assimilation. Atmospheric acidity has the potential to increase *δ*^15^N–NO_3_^−^ (ref. 32); however, spatial analyses of precipitation *δ*^15^N–NO_3_ from across the midwestern and eastern USA suggest that *δ*^15^N–NO_3_ is more influenced by source than precipitation pH, or SO_4_^2−^ or NO_3_^−^ concentrations^25^. Furthermore, paleolimnological data from our Uinta Mountain sites supports only a minor role for atmospheric acidity. These data show the most rapid decrease in lake sediment *δ*^15^N beginning in the mid-1900s and continuing to the present^16^, whereas reductions in atmospheric pH and SO_4_^2−^ or NO_3_^−^ concentrations show slight declines after 1985 at western US sites^33^.

During denitrification concentrations of NO_3_^−^ decrease (as NO_3_^−^ is converted to N_2_), *δ*^15^N–NO_3_ and *δ*^18^O–NO_3_ increase^34^, and *Δ*^17^O–NO_3_ remains unaffected. Although these features are observed in our study (Fig. 2), anaerobic conditions favouring denitrification are unlikely in the waters of oligotrophic Uinta Mountain lakes^16^,^35^. Moreover, previous studies suggest that 1:1 to 2:1 enrichment in ^15^N relative to ^18^O characterizes residual nitrate following denitrification^23^, whereas we observe a greater enrichment of ^18^O relative to ^15^N.

Differences in nitrate sources could also explain the higher *δ*^15^N–NO_3_^−^ in lakes than inflows. The variations in *δ*^15^N and *Δ*^17^O for both lake and inflow nitrates are small (Figs 2 and 3) considering the large range in nitrate concentrations (Fig. 2), site locations, inflow routes (for example, wetland and talus slopes) and sampling times (Supplementary Table 1). Given the many potential pathways travelled by NO_3_^−^ and processes affecting NO_3_^−^, such a small amount of variation is perhaps unexpected but is explained if source compositions are important in determining *δ*^15^N–NO_3_. This observation suggests the dominance of a widespread and isotopically homogenous source. This is supported by nearly identical AON source contribution to Uinta Mountain lakes and inflows as determined using *Δ*^17^O–NO_3_^−^ (MPEs of 23 and 24%, respectively) (Fig. 5). Snow samples, by comparison, have significantly higher *δ*^18^O- and *Δ*^17^O–NO_3_^−^ than lakes and inflows (Fig. 2), which is expected, because the isotopic composition of snow should be most connected to the atmosphere and least affected by the surrounding terrestrial system.

The AON originates mostly from fossil fuel combustion, lightning, soil emissions and biomass burning (Table 2). Based on global approximations of tropospheric NO_*x*_ sources^36^, we estimate that ∼10% of total nitrate inputs to Uinta Mountain lakes are derived from fossil fuel combustion, delivered as AON (Table 2). This may be an underestimation, as our sites lie upwind of two major sources of NO_*x*_, oil and gas production to the southwest in the Uinta Basin^37^ and the populous Wasatch Front to the west^38^. In addition, peroxyacetyl nitrates formed by reactions of fossil fuel-derived NO_2_ with peroxacyl radicals, themselves formed by oxidation and photolysis of volatile organic trace gases, could enable long-distance transport of NO_*x*_, enhancing delivery of AON to the Uinta Mountains from these upwind sources^39^.

The outstanding question is the source for the rest of the nitrate inputs. The lower *δ*^15^N–NO_3_^−^ of Uinta Mountain lakes and inflows indicates that a high proportion of the nitrate is derived from fertilizer (35% MPE for lakes and 62% MPE for inflows: NH_4_^+^ and NO_3_^−^ Fertilizer+Rain NH_4_^+^ in Table 1). The difference between lakes and inflows in apportionment of the NH_4_^+^ and NO_3_^−^ Fertilizer+Rain NH_4_^+^ (35% MPE versus 62% MPE) and Soil NO_3_^−^ categories (33% MPE versus 14% MPE) (Fig. 5 and Table 1) is initially surprising. As most water in lakes and inflows originates from precipitation, source apportionment should be similar, as is the case for the well-constrained AON contributions (Fig. 5). However, if additional Soil NO_3_^−^ in lakes originates from the catchment, then the AON contribution to lakes should have been lower than for inflows.

Nitrate assimilation in lakes can provide an explanation for their higher *δ*^15^N–NO_3_^−^ and lower NO_3_^−^ concentrations relative to inflows, and differences in source apportionment between lakes and inflows (Fig. 5). During the spring/summer periods sampled here, snowmelt results in peak nitrate inputs, which lead to increased lake production and thus nitrate assimilation^16^,^40^. During assimilation, lake NO_3_^−^ concentrations should decrease substantially relative to inflows, as is reported here (Fig. 2). Assimilation would also cause lake *δ*^15^N–NO_3_^−^ to increase, because algae and aquatic plants preferentially use ^14^N (ref. 41). Such ^15^N enrichment would cause the Soil NO_3_^−^ source category to be overrepresented in lakes relative to inflows, as is observed here.

Fertilizers, therefore, are currently the most important influence on nitrate concentrations in high elevation sites in the Uinta Mountains (Fig. 5). As neither NH_4_^+^- nor NO_3_^−^-based fertilizers are used directly in the catchments, this contribution is attributed to atmospherically delivered fertilizers from nearby to distant agricultural regions. Based on the main wind directions being south, southwest and west^42^,^43^,^44^, the most probable source is the Wasatch Front, but it could be much further away (for example, California) (see Supplementary Methods for additional information on source areas).

Collectively, these arguments lead to the conclusion that anthropogenic sources (primarily atmospherically delivered fertilizers (∼60%) and fossil fuel combustion (∼10%)) currently comprise ∼70% MPE of the nitrates delivered to the Uinta Mountain alpine aquatic ecosystems. A sizeable fraction of this nitrate is being assimilated by Uinta Mountain lakes, with attendant implications for increased primary lacustrine productivity.

Can we extend these findings to other western alpine sites? The average snow *Δ*^17^O–NO_3_^−^ from the Uinta Mountains (+23.7±5.6‰) is within the documented atmospheric range in non-polar regions of +24 to +33‰ (ref. 45) and comparable to the average *Δ*^17^O–NO_3_^−^ of three samples from a small catchment in the Colorado Front Range (+28.6±0.2‰)^46^. Considerable overlap also exists between Uinta Mountain snow nitrates (*δ*^18^O–NO_3_^−^ range: +48.6 to +75.1‰, median: +69.4‰) and precipitation nitrate measured in several US Cordilleran national parks (*δ*^18^O–NO_3_^−^ range: +71 to +78‰, median: +74.5‰)^30^. Similarly, high *δ*^18^O*–*NO_3_^−^ in several US Cordilleran lakes^30^ shows considerable overlap with Uinta Mountain lake nitrates (Fig. 6). The high *δ*^18^O–NO_3_^−^ (>0‰) and low *δ*^15^N–NO_3_^−^ (<+5‰) of these US Cordilleran lakes suggest influence by wet deposition of dissolved inorganic nitrogen (NO_3_^−^+NH_4_^+^) and fertilizer sources of dissolved inorganic nitrogen^30^. In the absence of *Δ*^17^O–NO_3_ data for other alpine lakes, this comparison suggests widespread atmospheric inputs of oxidized nitrate to alpine settings in western North America and potential effects from such nitrogen fertilization.

Baseline (that is, before fossil fuel burning and synthetic fertilizer production) nitrate concentrations remain virtually unknown for North American alpine sites. In the absence of such information, our results provide evidence for proportionally large contributions of nitrogen from new, anthropogenic sources, most importantly from atmospheric deposition of fertilizers used in agriculture. Based on paleolimnological evidence, it has been suggested that increased nitrogen delivery from these additional sources began at the onset of the twentieth century but was most pronounced after the mid-twentieth century^47^. Increased nitrogen inputs have led to increased lake primary production and other ecological changes in the Colorado Front Range^24^,^48^, Beartooth Range^49^ and Uinta Mountains^16^. Our study confirms these new sources of nitrogen. Taken together, and assuming that human populations continue to expand and agricultural fertilizer use continues to rise, these findings point to potential future of N-enrichment, decreased water quality and loss of biodiversity. The findings of our research, achieved by combining measurement of nitrate *Δ*^17^O, *δ*^18^O and *δ*^15^N with a Bayesian mixing model to discriminate among Nr sources, point to the challenges faced by society to balance needs for adequate food production with protection of critical water resources needed by our rapidly expanding population.

## Methods

### Study lake selection

Seven high elevation (>3,000 m.a.s.l.), oligotrophic lakes were selected for this study from the Uinta Mountains. These study lakes, with lake codes used in this study shown in bold and Utah Department of Wildlife Resources codes in parentheses, are: Denise **UN07** (WR-9), Taylor **UN08** (WR-8), Upper Carrol **UN55** (X-18), East Carrol **UN56** (X-21), No Name **UN57** (X-26), Bluebell Pass **UN58** (X-25) and Walk-Up Lake **UN32** (WR-55) (Fig. 1). Lake water, inflow and snow samples were collected from each site in summer 2008, 2009 and 2012 (Supplementary Table 1). Lake and inflow samples represent a single day sample and therefore a snapshot view, whereas the snow samples represent seasonal accumulation. To evaluate the effect on the isotopic composition that could occur during snowmelt, snow samples were also collected around the time of maximum snow accumulation but before spring snowmelt (March 2009 and April 2011). For logistical reasons, snow sampling at the time of maximum snow accumulation was not possible at the lake sites, but was performed instead at nearby sites (Chepeta, Spirit Lake and Lake Fork sites are within 13 km of the lake sites) and at the west and east extent of the Uinta mountains (Trial Lake and Grizzly Ridge sites are within 50 km of the lake sites) (Fig. 1) with the support of the United States Department of Agriculture and the United States Geological Survey.

### Water and snow sampling

For the lake water samples, the bottles were filled ∼0.5 m below the water surface at the deepest portion of the lake. Inflow samples were collected from streams and in some cases from rivulets from melting snow. Samples were collected for stable isotope analysis in 250 ml amber Nalgene bottles, kept cool in the field and frozen as soon as logistically possible (1–3 days). Summer snowpack samples were taken in zip lock bags after removing the outer ∼2 cm of surface snow. The snow samples were transferred to tightly sealed sample bottles in the field after melting and then frozen. Samples for stable isotope measurements were stored frozen until analysed at the Laboratory for Stable Isotope Science (LSIS), located at the University of Western Ontario in London, Canada.

The March 2009 snow samples (Lake Fork, Spirit Lake, Chepeta Lake and Trial Lake sites) were collected by the Natural Resources Conservation Services of the United States Department of Agriculture with a standard federal snow sampler using standard snow sampling techniques^50^. The April 2011 samples (Grizzly Ridge and Lake Fork sites) were collected from snow pits following methods used by the United States Geological Survey^51^. The samples were shipped frozen in clean, 19-l paint buckets to Lakes and Reservoir Systems Research Facility, also at the University of Western Ontario, where they were melted and aliquots provided to LSIS for stable isotopic analysis. Samples for water chemistry analysis (NO_2_, NH_4_ and NO_2_+NO_3_) were taken concurrently with the isotope samples and filtered using a 0.7-μm Whatman GF/F filter. Samples were frozen and sent to Chesapeake Biological Laboratory in MD, USA, for chemical analyses.

Of the collected inflow, lake and snow samples, 34 had sufficiently high nitrate concentrations (>1.6 μM) for analysis of *δ*^15^N-, *δ*^18^O- and *Δ*^17^O–NO_3_^−^. As only two lake samples met this nitrate concentration threshold, we analysed ten previously excluded lake samples (owing to nitrate concentrations <1.6 μM NO_3_). Of these, we were able to obtain *δ*^15^N and *δ*^18^O for seven samples, three of which also yielded *Δ*^17^O data. In total, 41 Uinta Mountain samples and one sample from Great Salt Lake (see Supplementary Methods) were analysed for stable isotopes of nitrate (Supplementary Table 1).

### Nitrate isotope analysis

We used the coupled cadmium-azide reduction method with some modifications to prepare nitrate for analysis of *δ*^18^O, *Δ*^17^O and *δ*^15^N. The chemical procedure involves two main steps. The first is conversion of nitrate to nitrite using activated cadmium and the second is conversion of nitrite to N_2_O by injecting 0.8 ml of a 1:1 by volume mixture of 2 M sodium azide and 20% acetic acid^52^. The *δ*^15^N and *δ*^18^O values are measured from N_2_O by monitoring the masses 44, 45 and 46 (^14^N^14^N^16^O, ^14^N^15^N^16^O+^14^N^14^N^17^O and ^14^N^14^N^18^O, respectively). The *Δ*^17^O values are measured on O_2_ formed by thermal decomposition of N_2_O using a gold catalyst at 875 °C (ref. 53) by monitoring the masses 32, 33 and 34. All measurements were made using a Thermo Finnigan Delta^PLUS^ XL isotope ratio mass spectrometer accessorized with a Gas Bench II and CTC CombiPal autosampler.

### Corrections and calibration

A number of corrections are made to the raw *δ*-values to account for overlapping masses and memory effects^54^. Briefly, for *δ*^15^N, two corrections are made to account for the mass overlap between ^15^N^14^N^16^O and ^14^N^14^N^17^O (mass=45). First, a ‘Craig Correction' is applied by the isotope ratio mass spectrometer operating system (ISODAT) and accounts for the mass overlap of these isotopologues. Second, a correction is applied to account for the mass-independent (that is, *Δ*^17^O>0) overlap between these same isotopologues, for which the Craig correction does not account. Through experimentation, we have determined that uncorrected *δ*^15^N is increased by 0.1‰ (denoted *X*_corr_ below) for every 1‰ increase in *Δ*^17^O. This offset in *δ*^15^N was determined from equation (1):


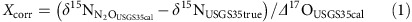


where *δ*^15^N_N_2_O__USGS35cal___, *δ*^15^N_USGS35true_ and *Δ*^17^O_USGS35cal_ are the calibrated (cal) and true values of USGS-35 nitrate. To account for the non-zero *Δ*^17^O, we have applied equation (2):


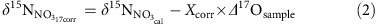


where 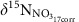 is the corrected value reported in this study, 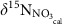 is the calibrated result produced using standards and *Δ*^17^O_sample_ is the *Δ*^17^O value of the sample. Therefore, for snow samples with *Δ*^17^O upwards of +30‰, this effect causes uncorrected *δ*^15^N to be higher than the true value by ∼+3‰. In the case of four lake water samples, we could not correct for the mass overlap between ^15^N^14^N^16^O and ^14^N^14^N^17^O, because the nitrate concentrations were too low to measure *Δ*^17^O accurately. In these cases, uncorrected *δ*^15^N–NO_3_^−^ was used. Although the inclusion of uncorrected results is not ideal, the correction would have probably been minor, because the lakes tend to have low *Δ*^17^O–NO_3_^−^ (mean lake *Δ*^17^O=+5.3‰). Corrections are also made to *δ*^18^O in each analytical run to account for exchange between sample water and laboratory water. This is necessary, because a fraction (on average 13%) of oxygen that ends up in the measured nitrous oxide product is derived from exchange with water oxygen rather than from the original nitrate oxygen.

Calibration of the raw isotopic ratios to AIR (nitrogen) and VSMOW (Standard Mean Ocean Water; oxygen) is achieved using international standards USGS-32, USGS-34 and USGS-35 (Supplementary Table 3), and an internal standard GSI-NO-3 (*δ*^15^N=+1.3‰, *δ*^18^O=+14.13‰, courtesy of the Geological Survey of Israel). Accuracy of isotopic compositions was determined by comparison with the value known for standard IAEA-NO-3. Mean *δ*^18^O, *Δ*^17^O and *δ*^15^N obtained on replicate measurements (*n*=10) of IAEA-NO3 are summarized and compared with accepted values^54^ in Supplementary Table 3. Precision was calculated using duplicates from each analytical session (±0.04‰, ±0.23‰ and ±0.5‰ for *Δ*^17^O, *δ*^18^O and *δ*^15^N, respectively). Internal data from LSIS (*N*=48) has demonstrated long-term precision for dissolved nitrate sample duplicates to be ±0.50‰, ±1.00‰ and ±0.7‰ for *Δ*^17^O, *δ*^18^O and *δ*^15^N, respectively.

### Stable isotopes analysis in R mixing model

Proportional contributions of nitrate sources to Uinta Mountain lakes and inflows were estimated using the stable isotope mixing model SIAR^15^. SIAR uses a Bayesian framework, to determine the probability distribution of the proportional contribution of various sources to a mixture. The mathematical definition used can be found in the Supplementary Methods. The model is fit via Markov chain Monte Carlo methods, which produces simulations of plausible source proportions for each sample group^15^. Models were run for 500,000 iterations. In this study, we used both *δ*^15^N and *Δ*^17^O in the SIAR model, to differentiate contributions from four source categories: AON, NH_4_^+^ and NO_3_^−^ Fertilizer+Rain NH_4_^+^, Soil NO_3_^−^ and Septic Effluent and Manure. Source parameters (mean and s.d.) for input into the SIAR model were estimated from ranges provided in the literature^21^ and are listed in Supplementary Table 2. Two snowmelt samples (Fig. 3) were not included in the SIAR model, because they were located directly below snowpack and were not clearly categorized into either the snowpack or inflow sample group. These data nonetheless provide information on transitional waters between snow and stream. The four lake samples for which we were unable to measure *Δ*^17^O were also not included in the SIAR model. We used the mean and s.d. of all Uinta Mountain snow sample nitrate isotope compositions (mean *Δ*^17^O–NO_3_^−^ is +23.7±5.6‰) to represent the regional AON source; this value is comparable to the range in *Δ*^17^O–NO_3_^−^ observed in non-polar regions (+24 to +33‰)^45^. We acknowledge that only winter precipitation is represented in our sample set, and that atmospheric *Δ*^17^O can vary seasonally^55^, but felt that our snow sample average was a more accurate representation of regional AON values than a global average.

After initial model runs, which contained five possible nitrate sources (NO_3_^−^ Fertilizer was initially separate from NH_4_^+^ Fertilizer+Rain NH_4_^+^) and *δ*^15^N-, *δ*^18^O- and *Δ*^17^O–NO_3_^−^ measurements, we simplified the model in two ways. First, NO_3_^−^ Fertilizer and NH_4_^+^ Fertilizer+Rain NH_4_^+^ were aggregated into the single source described earlier and, second, the model inputs were reduced from three isotopes of nitrate to include only *δ*^15^N and *Δ*^17^O. We combined the NO_3_^−^-based fertilizer and NH_4_^+^-based fertilizer+rain NH_4_^+^ source values into a single source, because they have similar nitrogen isotopic signatures. In addition, both NO_3_^−^-based and NH_4_^+^-based fertilizers are associated with intensive agricultural activity. NH_4_^+^ in rain is also largely derived from fertilizer, although natural soil emissions and manure can also contribute^21^,^29^. Given that *Δ*^17^O- and *δ*^18^O–NO_3_^−^ are highly correlated (*R*^2^=0.94) and both tracking AON, we removed the parameter *δ*^18^O–NO_3_^−^ from the models. This strengthens the model, as the potential variability in *δ*^18^O for terrestrial nitrate and atmospheric nitrate sources is much larger than for *Δ*^17^O. It is not possible to predict the correlation of *Δ*^17^O and *δ*^18^O without making measurements of both. If samples consisted of primarily terrestrial nitrates, the samples would track the terrestrial fractionation line, but this behaviour cannot be discerned from *δ*^18^O alone.

### Estimation of snow *δ* ^15^N–NH_4_^+^

A large component of snow N is in the form NH_4_^+^, but our analysis solely measures *δ*^15^N–NO_3_^−^. To improve our understanding of the source values of NH_4_^+^ in snow, we can calculate the approximate value of *δ*^15^N–NH_4_^+^. Assuming that the inflow *δ*^15^N reflects the combined isotopic composition of snow ammonium and nitrate, the *δ*^15^N of snow NH_4_^+^ is calculated by solving for *δ*^15^N_NH_4_^+^snow_ in equation (3):


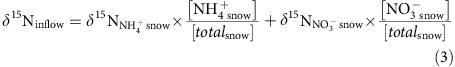


where *δ*^15^N_inflow_ and *δ*^15^N_snow_ are average values. Both NH_4_^+^ and NO_3_^−^ are reported as N in μM, [*total*_snow_] is the sum of [NH_4 snow_^+^] and [NO_3 snow_^−^], and *δ*^15^N_NO_3_^−^snow_ is assumed to be equal to *δ*^15^N_snow_ (for data, see Supplementary Table 1). This calculation also assumes that there is little fractionation during conversion from ammonium to nitrate.

## Additional information

**How to cite this article:** Hundey, E. J. *et al*. Agriculture causes nitrate fertilization of remote alpine lakes. *Nat. Commun.* 7:10571 doi: 10.1038/ncomms10571 (2016).

## Supplementary Material

Supplementary Information Supplementary Tables 1-3, Supplementary Methods and Supplementary References.

## Figures and Tables

**Table 1:** SIAR model results for various sources of nitrate to inflows and lakes.

**Group**	**Source**	**MPE**	**Low 95% highest density region**	**High 95% highest density region**
Inflows	AON	23	20	26
	NH_4_^+^ and NO_3_^−^ Fertilizer+Rain NH_4_^+^	62	49	74
	Soil NO_3_^−^	14	1	26
	Septic Effluent and Manure	1	0	5
Lakes	AON	24	14	38
	NH_4_^+^ and NO_3_^−^ Fertilizer+Rain NH_4_^+^	35	14	55
	Soil NO_3_^−^	34	5	55
	Septic Effluent and Manure	2	0	23

MPE, modal probability estimate.

The MPE is the solution (proportion contributed by that source) with the highest probability. The low 95% and high 95% highest density region encompass 95% of the model solutions.

**Table 2:** Sources of tropospheric NO_*x*_ and their estimated contribution to Uinta Mountain lake nitrates.

**Source of tropospheric NO**_***x***_	**Contribution (Tg** **N** **per year) to tropospheric NO**_***x***_	**Contribution (%) to tropospheric NO**_***x***_	**Estimated contribution to Uinta Mountain lakes (% total nitrate)**
Fossil fuels	24	40	10
Lightning	12	20	5
Soil emissions	12	20	5
Biomass burning	8	13	3
NH_3_ oxidation	3	5	1
Transport from stratosphere	0.4	0.7	0.2
Aircraft	0.4	0.7	0.2
Total	60	100	24

AON, atmospherically oxidized nitrate; MPE, modal probability estimate.

The estimated contribution of each tropospheric NO_*x*_ source to Uinta Mountain lakes is calculated based on the global percentage contribution to the troposphere^36^ and an overall contribution of AON to Uinta Mountain lakes of 24% MPE.
