# Supplementary material for: Agriculture causes nitrate fertilization of remote alpine lakes
Source: Nat Commun. 2016 Feb 8;7:10571. doi: 10.1038/ncomms10571 (PMC4748117; doi:10.1038/ncomms10571)
Supplement: Supplementary Information — Supplementary Tables 1-3, Supplementary Methods and Supplementary References. [file ncomms10571-s1.pdf]

Supplementary Table 1. Water sample data from triple isotope analysis of nitrates.

Date MM/YY	Sample Name	Sample Type	Latitude (N)	Longitude (W)	Elev. (m)	δ^{15}N ‰ AIR	δ^{18}O ‰ VSMOW	Δ^{17}O ‰ VSMOW	NO_3^- (μM)	NH_4^+ (μM)
03/09	Chepeta	snow	40.76	110.016	3228	+1.5	+75.1	+30.7	7.85	5.78
03/09	Trial Lk	snow	40.68	110.95	3046	+0.6	+71.7	+25.3	4.38	4.64
03/09	Spirit Lk	snow	40.83	110.0	3116	+2.1	+74.1	+29.2	10.14	7.43
03/09	Lakefork	snow	40.6	110.43	3174	+3.4	+73.8	+28.5	7.50	6.57
04/11	Lakefork	snow	40.5955	110.4339	3094	+1.8	+55.0	+20.2	10.00	6.0
04/11	Grizzly Ridge	snow	40.7489	109.5051	2914	–1.2	+48.6	+15.0	15.79	3.4
07/09	UN58SNOW	snow	40.7117	110.3923	3611	–0.8	+62.5	+20.0	2.59	4.64
08/09	UN08SNOW	snow	40.7904	110.0954	3486	0.0	+67.0	+20.3	2.01	8.07
07/08	UN08IN1A	snowmelt	40.7904	110.0953	3483	+2.7	+52.5	+17.1	1.86	3.00
07/08	UN08IN1B	snowmelt	40.7904	110.0952	3469	–3.7	+35.8	+12.7	4.31	0.79
07/08	UN08IN1C	inflow	40.7899	110.0935	3428	–1.2	+11.9	+5.8	19.78	0.78
07/08	UN08IN1D	inflow	40.7902	110.0939	3435	–2.0	+14.7	+7.4	14.79	0.71
07/08	UN08IN2	inflow	40.7888	110.0939	3429	–3.3	+13.4	+6.2	28.47	0.64
07/08	UN08IN3	inflow	40.7876	110.0931	3426	–1.4	+7.3	+4.4	9.20	1.21
07/08	UN07IN	inflow	40.7820	110.0872	3408	–1.3	–4.7	+0.5	2.88	0.64
07/08	UN55IN1	inflow	40.7226	110.3564	3403	–0.7	+9.5	+4.5	14.69	0.57
07/08	UN56IN1	inflow	40.7198	110.3451	3428	–2.0	+11.4	+5.0	41.39	0.57
07/08	UN58IN1A	inflow	40.7006	110.3881	3366	–2.6	+17.1	+7.2	47.18	0.64
07/08	UN58IN1B	inflow	40.7009	110.3877	3354	–2.8	+17.8	+6.9	46.50	0.71
08/09	UN08IN1A	inflow	40.7907	110.0943	3447	–0.9	+11.2	+5.4	19.92	0.92
08/09	UN08IN1B	inflow	40.7902	110.0938	3434	–1.3	+11.3	+5.5	19.42	0.64
08/09	UN08IN1C	inflow	40.7921	110.0948	3456	–2.1	+15.3	+6.9	15.14	0.57
08/09	UN08IN2	inflow	40.7888	110.0939	3432	–1.6	+12.3	+5.9	55.97	0.86
08/09	UN08IN3	inflow	40.7876	110.0931	3426	–0.9	+10.4	+4.8	38.98	0.57
08/09	UN07IN1	inflow	40.7820	110.0872	3408	–1.3	–2.4	+1.8	6.20	1.14
07/09	UN55IN1A	inflow	40.7226	110.3564	3403	–1.1	+13.0	+5.9	19.42	1.00
07/09	UN55IN1B	inflow	40.7230	110.3576	3415	+1.0	+13.1	+5.1	8.21	4.36
07/09	UN56IN1A	inflow	40.7203	110.3444	3435	–2.0	+13.1	+5.1	45.12	0.50
07/09	UN56IN1B	inflow	40.7198	110.3451	3428	–1.6	+13.1	+4.9	44.48	0.71
07/09	UN58IN1A	inflow	40.7006	110.3881	3366	–1.7	+15.9	+6.8	44.55	0.79
07/09	UN58IN1B	inflow	40.7006	110.3881	3364	–2.0	+16.2	+6.3	45.19	0.57
07/09	UN58IN1C	inflow	40.7009	110.3877	3353	–2.7	+15.6	+6.6	44.55	0.64
07/08	UN32	lake	40.8114	110.0380	3393	+2.4	+12.3	+4.7	7.35	0.50
07/08	UN08A	lake	40.7884	110.0926	3424	+0.4	+15.6	+2.6	1.83	0.93
07/08	UN55	lake	40.7215	110.3545	3400	–1.1	+24.4		0.39	0.50
07/08	UN58	lake	40.7017	110.3866	3400	+0.0	+21.6		0.76	0.50
07/09	UN56	lake	40.7187	110.3449	3424	+0.4	+14.6	+3.8	0.78	1.57
08/09	UN08b	lake	40.7884	110.0926	3425	+0.9	+18.0		0.21	0.79
08/09	UN07	lake	40.7818	110.0860	3404	+2.3	+12.4		0.59	1.21
05/12	UN55	lake	40.7215	110.3545	3400	+1.5	+9.6	+8.0	0.87	1.43
05/12	UN08	lake	40.7884	110.0926	3424	+0.8	+21.9	+7.5	1.57	0.64
07/11	GSLH2O	GSL	40.7357	112.2107	1281	+10.4	+16.3	+4.7	3.06	
Snow mean and SD (n=8)						+0.9±1.6	+66.0±9.9	+23.7±5.6	7.5±4.6	5.8±1.6
Lakes mean and SD (n=9)*						+0.8±1.1	+16.7±5.1	+5.2±2.1	1.6±2.2	0.9±0.4
Inflows mean and SD (n=22) †						–1.6±0.9	+11.7±5.5	+5.4±1.7	28.7±16.7	0.9±0.8

*for lake Δ^{17}O, n=5, due to low concentrations of nitrate in 4 lake samples.

†mean and SD reported are based on 22 inflow samples without the two snowmelt samples, which are not used in the SIAR model.

Supplementary Table 2. SIAR input values for nitrate sources.

Source	$\delta^{15}N$ ‰		$\Delta^{17}O$ ‰	
	Mean	SD	Mean	SD
AON	+0.94	1.57	+23.66	5.58
NH_4^+ and NO_3^- Fertilizer + Rain NH_4^+	–1.4	5.1	0	1
Soil NO_3^-	+4.15	3.4	0	1
Septic Effluent and Manure	+16.05	12.75	0	1

Source values for "NH_4^+ and NO_3^- Fertilizer + Rain NH_4^+", "Soil NO_3^-", and "Septic Effluent and Manure" were estimated from ranges provided in the literature[1]. Atmospherically oxidized nitrate ("AON") source values were calculated based on Uinta Mountain snow samples.

Supplementary Table 3. Standard nitrate isotope compositions for calibration.

		**$\delta^{15}N$ (‰$_{AIRN2}$)**	**$\delta^{18}O$ (‰$_{VSMOW}$)**	**$\Delta^{17}O$ (‰$_{VSMOW}$)**
International Standards	USGS-32	+180 ± 1	+ 25.7 ± 0.4	Unknown
	USGS-34	–1.8 ± 0.2	–27.90 ± 0.6	–0.3
	USGS-35	+2.7 ± 0.2	+57.50 ± 0.6	21.60
Precision	IAEA-NO-3 Accepted value	+4.7 ± 0.2	+25.60 ± 0.4	–0.10
	IAEA-NO-3 Check	+4.8 ± 0.1 (n=10)	+25.49 ± 0.11 (n=10)	–0.48 ± 0.21 (n=10)

USGS-32, USGS-34, and USGS 35 are international standards used for calibration (±1 standard deviation) and IAEA-NO-3 is used to assess measurement accuracy. The $\delta^{15}N$ and $\delta^{18}O$ values are obtained from www.nucleus.iaea.org. The $\Delta^{17}O$ values are drawn from the literature[2].

Supplementary Methods

One potential source of precipitation and nitrogen to the Uinta Mountain lakes is Great Salt Lake, a large (4400 km^2) body of water located upwind (Fig. 1). A water sample was obtained from Great Salt Lake in July 2011 in order to compare nitrate isotopic compositions originating from this large, saline, polluted lake with the compositions of nitrates in the Uinta Mountains. Although we considered it unlikely that Great Salt Lake would contribute significantly to the nitrate pool in the Uinta Mountains, we analyzed the sample to test, and ultimately exclude, this possibility. Great Salt Lake nitrate has similar $\Delta^{17}O$- and $\delta^{18}O\text{-}NO_3^-$ to the Uinta Mountains but has much higher $\delta^{15}N\text{-}NO_3^-$ (Supplementary Table 1). This result suggests that Great Salt Lake nitrate originates from septic and manure inputs[3] and makes little to no contribution to Uinta Mountain nitrate. Moreover, the $\delta^{15}N$ and $\Delta^{17}O$ of nitrates that originated from Great Salt Lake could be increased via denitrification in Great Salt Lake and in combination with ozone in the atmosphere, respectively. Since the isotopic compositions in the Uinta Mountains are not indicative of these processes, it is unlikely that Uinta Mountain lakes receive much, if any, Nr originating from Great Salt Lake.

Based on the main contributor of Nr being fertilizer, we considered potential geographical sources of atmospheric Nr. To do this we found the best available wind data and considered potential source distances based on residence times[4] (Wasiuta et al., 2015a). Based on residence times, Waisuta et al. (2015b)[5] classify potential sources as being local (within a half day travel or 50-400 km), regional (within 2 days travel or 290-680 km), or distances greater than 680 km, and indicate that sources to their sites could be as much as 725-1000 km. This led us to suggest two potential geographical

sources of fertilizer inputs – the Wasatch Front and California, but future research should be undertaken to pinpoint the geographical source(s).

The mathematical definition of SIAR employed in this paper is given below. It is modified from the original[6] by the exclusion of concentration factors that are not required for this application of SIAR to nitrate stable isotopic data[7]. The excluded functions were originally designed to account for differences in the concentration of carbon and nitrogen in different dietary sources.

$$X_{ij} = \sum_{k=1}^{K} p_k\left(s_{jk} + c_{jk}\right) + \varepsilon_{ij}$$

$$s_{jk} \sim N\left(\mu_{jk}, \omega_{jk}^2\right)$$

$$c_{jk} \sim N\left(\lambda_{jk}, \tau_{jk}^2\right) \tau$$

$$\varepsilon_{ij} \sim N\left(0, \sigma_j^2\right)$$

Definitions:

X_{ij} is the stable isotope composition value *j* of the sample group *i*

S_{jk} is the source value *k* on the isotope *j* with mean and variance (μ_{jk}, $\omega^2{}_{jk}$)

c_{jk} is the fractionation factor for the source *k* on the isotope *j* with mean and variance (λ_{jk}, $\tau^2{}_{jk}$)

ε_{jk} is the residual error that describes the variance not described by the model from a mean 0 and standard deviation $\sigma^2{}_J$

Supplementary References

1. Xue, D. *et al*. Present limitations and future prospects of stable isotope methods for nitrate source identification in surface- and groundwater. *Water Res.* **43**, 1159-1170 (2009).

2. Böhlke, J., Mroczkowski, S. & Coplen, T. Oxygen isotopes in nitrate: new reference materials for ^{18}O:^{17}O:^{16}O measurements and observations on nitrate-water equilibration. *Rapid Commun. Mass Sp.* **17,** 1835-1846 (2003).

3. Baskin, R.L. *et al*. Water quality in the Great Salt Lake basins: Utah, Idaho, and Wyoming, 1998-2001. U.S. Geological Survey Water Resources Investigations Report 02–4115 (2002).

4. Wasiuta, V., Lafrenière, M.J., Norman, A. & Hastings, M.G. Summer deposition of sulfate and reactive nitrogen to two alpine valleys in the Canadian Rocky Mountains. *Atmos. Environ.* 10, 270-285 (2015a).

5. Wasiuta, V., Lafrenière, M.J., Norman, A. Atmospheric deposition of sulphur and inorganic nitrogen in the Southern Canadian Rocky Mountains from seasonal snowpacks and bulk summer precipitation. *J. Hydrol*. 523, 563-573 (2015b).

6. Parnell, A.C., Inger, R., Bearhop, S., & Jackson, A.L. Source partitioning using stable isotopes. Coping with too much variation. *Plos One* **5,** e9672 (2010).

7. Xue, D., De Baets, B., Van Cleemput, O., Hennessy, C., Berglund, M., & Boeckx, P. Use of a Bayesian isotope mixing model to estimate proportional contributions of multiple nitrate sources in surface water. *Environ. Pollut.* **161,** 43-49 (2012).
